# Notice of Dr. Meigs’ Letters on the Diseases of Woman

**Published:** 1859-08

**Authors:** 


*Woman: Her Diseases and Remedies. A Series of Letters to his Class.* By Charles D. Meigs, M. D., Professor of Midwifery and the Diseases of Women and Children, in the Jefferson Medical College, at Philadelphia, etc. etc. Fourth edition, revised and enlarged. Philadelphia. Blanchard & Lea, 1859.

The letters to his class, by the eminent Professor of Midwifery at the Jefferson Medical College, are already so well known to the profession by the three preceding editions, that we have simply to make the announcement of a fourth. The peculiarities of the author's style have been abundantly commented upon by the medical press; and we are now entitled to pass that over in our notice of the work. Suffice it to say that, though somewhat abated in the new edition, the work is still marked by the peculiarities with which we are all so familiar.

